# Assessment of Radiation and Heavy Metals Risk due to the Dietary Intake of Marine Fishes (*Rastrelliger kanagurta*) from the Straits of Malacca

**DOI:** 10.1371/journal.pone.0128790

**Published:** 2015-06-15

**Authors:** M. U. Khandaker, Kh. Asaduzzaman, S. M. Nawi, A. R. Usman, Y. M. Amin, E. Daar, D. A. Bradley, H. Ahmed, A. A. Okhunov

**Affiliations:** 1 Department of Physics, Faculty of Science, University of Malaya, Kuala Lumpur, 50603, Malaysia; 2 Department of Physics, University of Surrey, Guildford, GU2 7XH, United Kingdom; 3 Department of Physics, Faculty of Science, University of Jordan, Amman, 11942, Jordan; 4 Physics Dept., Faculty of Science, Jazan University, Gizan, 22822, Kingdom of Saudi Arabia; 5 Department of Science in Engineering, International Islamic University Malaya, Kuala Lumpur, 50728, Malaysia; Dowling College, UNITED STATES

## Abstract

The environment of the Straits of Malacca receives pollution as a result of various industrial and anthropogenic sources, making systematic studies crucial in determining the prevailing water quality. Present study concerns concentrations of natural radionuclides and heavy metals in marine fish (*Rastrelliger kanagurta*) collected from the Straits of Malacca, since aquatic stock form an important source of the daily diet of the surrouding populace. Assessment was made of the concentrations of key indicator radionuclides (^226^Ra, ^232^Th, ^40^K) and heavy metals (As, Mn, Fe, Cr, Ni, Zn, Cu, Co, Sr, Al, Hg and Pb) together with various radiation indices linked to the consumption of seafish. The annual effective dose for all detected radionuclides for all study locations has been found to be within UNSCEAR acceptable limits as has the associated life-time cancer risk. The overall contamination of the sampled fish from heavy metals was also found to be within limits of tolerance.

## Introduction

While humans are daily exposed to external radiation from cosmic and terrestrial radiation, radionuclides in the sea may more typically contribute to internal exposure via ingestion. Most of the radioactive progeny within the ^238^U and ^232^Th natural decay chains are γ-emitters, forming a major source of external exposures [1]. Conversely, some of their decay products such as ^226^Ra, ^222^Rn, ^218^Po, ^210^Po, ^224^Ra, ^220^Rn, ^212^Bi etc. are alpha emitters, added to which are beta emitters such as ^234^Pa, ^214^Bi, ^228^Ac, ^212^Bi etc., with ^40^K (decaying by β¯) forming yet another means of internal exposure from natural sources. The levels of these can be enhanced through anthropogenic activities, further added to by artifical sources of radioactivity introduced into the environment [1–3].

In the marine environment, radioactivity is contributed to by the natural processes of weathering and mineral recycling of terrestrial rocks, seabed movement arising from undersea earthquakes and underwater volcanic activity. Given the enormous contact with various types of minerals and geological materials such as igneous rocks and ores which often contain elevated concentrations of natural radionuclides, consequently ^226^Ra (^238^U), ^228^Ra (^232^Th) decay series radionuclides are transferred to water through leaching action [4]. Anthropogenic activities such as combustion of fossil fuel, as for example from coal-fired power plants, production of natural gas and oil, and mining and processing of ores etc. are also known to enhance the naturally occurring radioactivity in the marine environment. In addition, there are anthropomorphic contributions from post-nuclear disposal of industrial and radioactive waste, underwater nuclear device tests, accidents including leaks from nuclear power plants and from reprocessing of spent nuclear fuel etc [2, 5–7].

Although radionuclides in ocean shows a complex behaviour (for instance, uranium is quite soluble in sea water while thorium is almost totally insoluble, radium and radon is soluble in water), they can be transferred in the marine environment in the following ways: dissolved in the seawater, attached to plankton suspended in the seawater and attached to sediment on the seabed and contaminated the marine organism, including fish, shell fish etc [8].

In addition to pathways from radioactivity in the marine environment into the human diet, a further concern is metal contaminants via consumption of marine products, with bioaccumulation leading to potential risks via long-term exposure[9–13]. Marine life can have considerable capability for bioaccumulationand biosorption (Biosorption is a property of certain types of inactive, dead, microbial biomass to bind and concentrate heavy metals from even very dilute aqueous solutions. It is particularly the cell wall structure of certain algae, fungi and bacteria which was found responsible for this phenomenon. Marine animal has tendency to burrow down in the bottom sea sediments and rocks, filtering on organic particles and algae along with tiny fishes and planktons, which may lead to increase the uptake of radioactive and heavy metals) of radionuclides and toxic/heavy metals from their surroundings, not least fish and shell fish relative to other marine life (e.g., molluscs, crustaceans, and fishes) [2, 5, 14–19]. Seafood (e.g., molluscs, crustaceans, and fishes) and their products can typically be one of the major sources of protein to populations in coastline regions, including those around the Straits of Malacca, one of the most important shipping lanes in the world, transporting about one-quarter of the world's traded goods [2, 5, 14, 16, 20, 21]. Approximately three million barrels of crude oil are shipped through the Straits daily, subjecting the sensitive marine environment to the threat of accidental oil spillage; over the 10-year period 1981–199, an average of two to three oil spill incidents per year were recorded in these waters [21]. The distinct possibility of release of large quantities of metal contaminants from different sources includes association with transportation, with increasing tanker traffic adding to the concern, to which one can add offshore oil and gas exploration, the operation of power plants and other industrial activities, agricultural activities and the waste streams of urbanization, all of which may pose a significant danger to human health due to the non-biodegradability and accumulation of metals in the food chain [20, 22]. The metals include copper, zinc and iron, all essential within both marine and human metabolism while some others such as the heavy metals: mercury, cadmium, arsenic and lead have no known role in biological systems [20]. Together with essential metals,non-essential ones also taken up from water and solid nutrient sources, and can accumulate in the tissues [23]. Metals like Cd, Hg, As, Pb, Cr, Se, Ni etc. have been commonly found in human diets and have been reported to be carcinogenic and /or mutagenic in a broad spectrum of animal studies and short-term test systems, adding to the concern about the contribution of these elements to human carcinogenesis [24, 25].

Distribution of radioactivity in seafood differs with respect to sites of origin and feeding habits [26]. With uptake in the human clearly depending on dietary habit, it is therefore of interest to note that the region of present research to have one of the highest marine fish consumption rates in the world, to the extent that information on radionuclide balance in seafish assumes proportionally greater importance [27].Information on bioaccumulation and distribution of natural radionuclides and heavy metals in seafish and sea water within the available literature is still lacking. Given the importance of such knowledge, the objective of present research to determine the concentrations of natural radionuclides and heavy metals in edible marine life caught in Straits water, the daily intake of these radionuclides and heavy metals, the ingestion dose and carcinogenic risk for the public residing in and around the coastal area of peninsular Malaysia.

## Materials and Methods

### Study area

No specific permissions were required for these field studies (locations/activities), because the studied field locations are open and we have collected the fish sample from the fisherman of the corresponding locations who are fishing in the studied locations. The field studies did not involve any protected species of fish. We have collected the most commonly consumed fish in Malaysia

**Fig 1:**
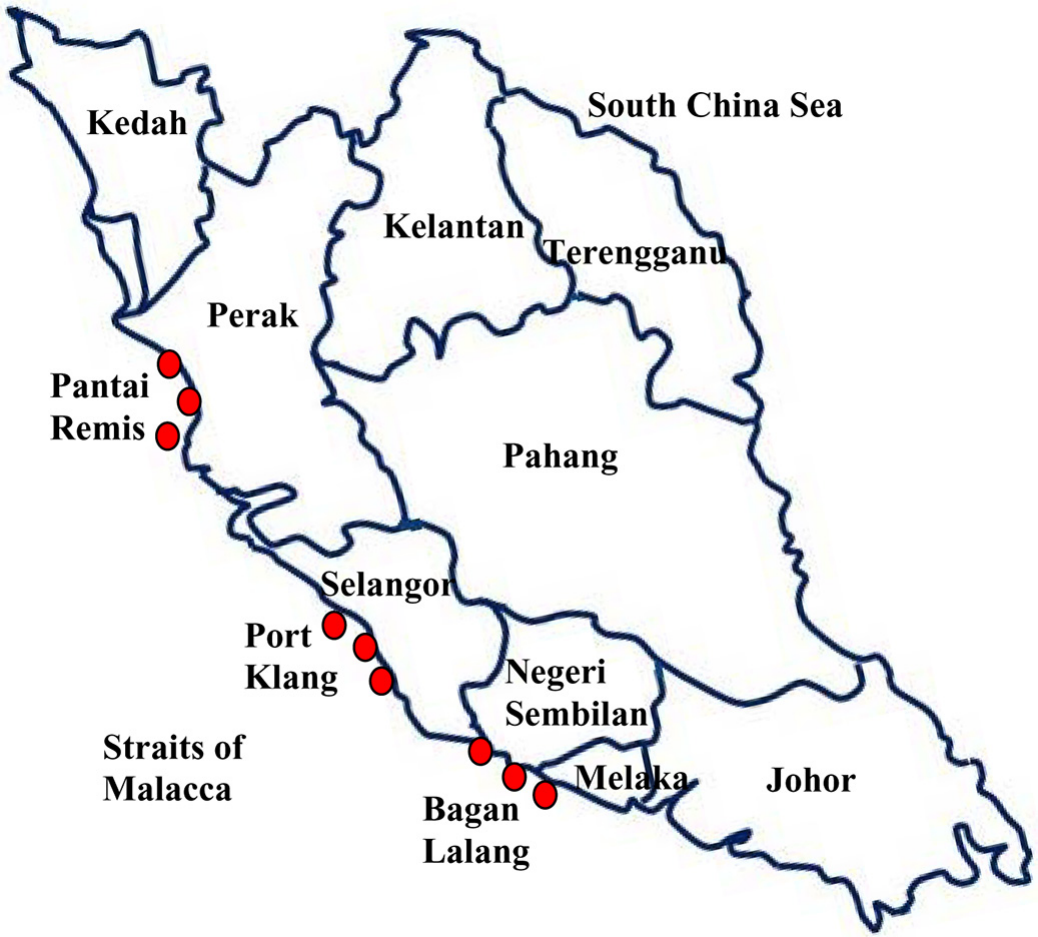
Location of marine fish sampling sites.

The primary fish landing areas of the west coast of Peninsular Malaysia, facing out to the Straits of Malacca, partitioned into three regions: the northern region (Perlis, Kedah, Penang and Perak), the central region (Selangor, Kuala Lumpur and Putrajaya) and the southern region (Negeri Sembilan, Malacca and Johor) were chosen as the sampling locations. Over the period November 2011 to February 2012, the samples were collected from coastal locations of Pantai Remis (4.4500° N, 100.6333° E) in Perak, Port Klang (3.0000° N, 101.4000° E) in Selangor, and Bagan Lalang (5.4333° N, 100.3833° E) in Negeri Sembilan, all surrounded by many fishing villages (Fig 1). In each case, there are nearby large-scale industrial activities, and a number of coal-, gas- and oil-fired power plants, including the 2295 MW Manjung coal-fired power plant in Perak, the 1400 MW Jimah coal-fired power plant in Negeri Sembilan and the 2420 MW Kapar coal-fired power plant in Klang, Selangor. Coal contains only a trace amount of radioactive uranium, barium, thorium and potassium, and are not known to create any major problem to the environment. However when coal is burned, in the fly ash that results, uranium and thorium are concentrated by up to 10 times their original levels [12, 28], and can be easily deposited and affacting the surrounding environment [29].

### Collection and processing of sample

**Fig 2:**
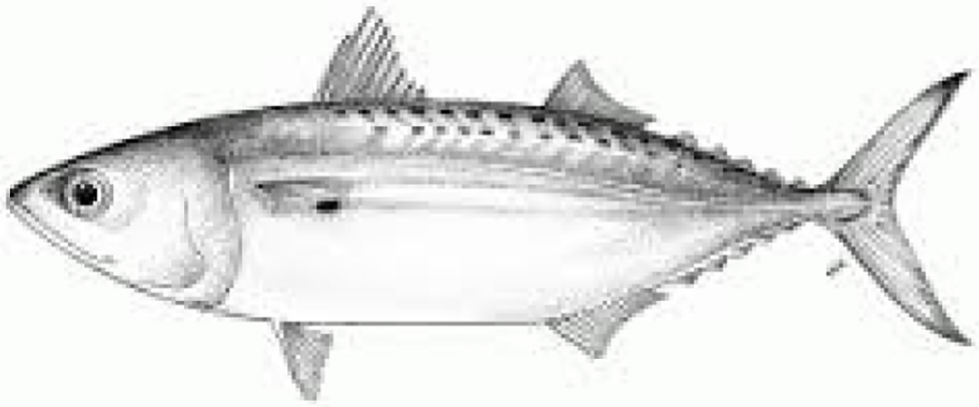
Kembung fish (*Rastrelliger kanagurta*).

The samples were of two categories: fish and seawater. The fish, a particular species of Mackerel (local and scientific name, Ikan kembung and *Rastrelliger kanagurta* (Phylum: Chordata; Family: Scombridae; Genus:*Rastrelliger;*Species:*R*. *kanagurta*) respectively)) were collected from coastal fishing jetties at various locations (Fig 1 and Table 1). The sampling informations are shown in Table 1. Same size and amount (125–130 g each) of fish samples (Fig 2) were collected andwashed, cut into smaller pieces and dried in a furnace at 70^°^C over a period of three days. The samples were then pulverized to obtain a fine powder and sieved for homogeneity. Sea water samples were collected from an approximate depth of one meter from the surface and filtered to remove any impurities. 120 to 218 g of each fish sample and 750 ml of each water sample were then sealed into Marinelli beakers and left for about 6–8 weeks to attain secular equilibrium between the ^238^U and ^232^Th precursors and their short-lived progenies.

**Table 1:** Marine fish samples collection data.

Local name (Scientific name)	Habitat	Diet	Sampling location (States)	GPS coordinate	Sample code	Number of sample	Measured length (cm)	Measured weight (g)
Ikan Kembung* (*Rastrelliger kanagurta*)	Pelagic (Warm shallow coastal waters)	Macroplankton including the larvae of shrimp and fish	Bagan Lalang (Negeri Sembilan)	(5.43333° N, 100.3833° E)	Fi_Bl-1	3	22–25	125–130
					Fi_Bl-2	3	22–25	125–130
					Fi_Bl-3	3	22–25	125–130
			Port Klang (Selangor)	(3.0000° N, 101.4000° E)	Fi_Kl-1	3	22–25	125–130
					Fi_Kl-2	3	22–25	125–130
					Fi_Kl-3	3	22–25	125–130
			Pantai Remis (Perak)	(4.500° N, 100.6333° E)	Fi_Re-1	3	22–25	125–130
					Fi_Re-2	3	22–25	125–130
					Fi_Re-3	3	22–25	125–130

*Indian mackerel

### Measurements of radioactivity

**Fig 3:**
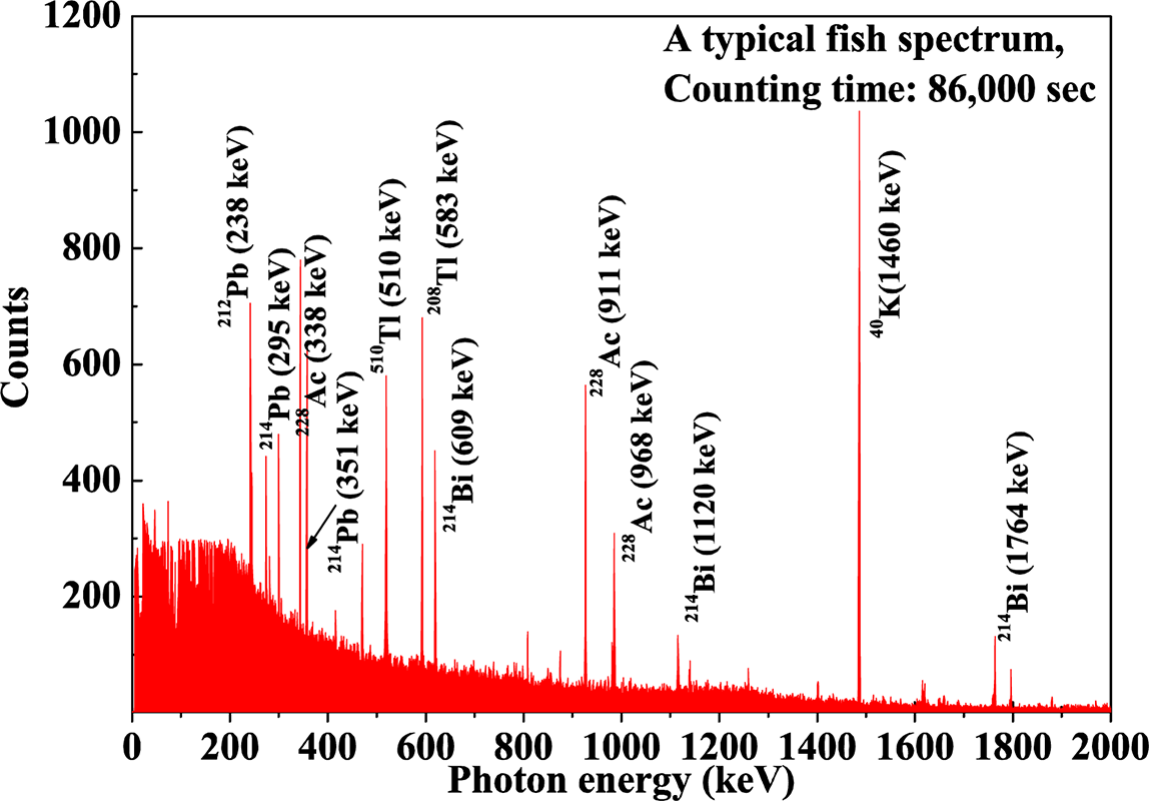
A typical gamma-ray spectrum collected from a fish sample.

The radioactivities of the samples were determined using a high resolution, p-type coaxial HPGe γ-ray spectrometer (ORTEC; GEM-25P; Serial no. 46-TP22121A; 57.5-mm crystal diameter and 51.5-mm thickness; operating voltage: +2800 V) shielded by cylindrical lead. The detector relative efficiency was 28.2% and energy resolution of 1.67 keV-FWHM at the 1.33 MeV peak of ^60^Co. The detector was coupled to a 16 k MCA to determine the photo-peak area of the γ-ray spectrum and analyzedby Gamma Vision 5.0 software (EG&G Ortec). A cylindrical multi-nuclide source was used for detector energy calibration and efficiency determination [30,31]. The measured detection efficiencies were fitted by using a polynomial fitting function as described in ref. [3], and the fitted efficiencies were used in activity determination of the samples. The minimum detectable activity (MDA) of the γ-ray measurement system at 95% confidence level was calculated according to the procedure in [32]. Each sample was counted for 86400 s and similarly for background counts to obtain the net activity. Fig 3 represents a typical gamma-ray spectrum collected from a fish sample. Only strong and independent characteristic gamma lines (the γ-rays highlighted in bold in Table 2) of the respective radionuclides were used to determine the net activity concentrations to reduce the error in activity determination.

**Table 2:** Decay data of the detected radionuclides of interest (Source: http://www.nndc.bnl.gov/nudat2/).

Radionuclides of interest	Detected radionuclides	Half-life	Decay mode (%)	γ-ray energy, E_γ_ (keV)	γ-ray intensity, P_γ_ (%)
^226^Ra	^214^Pb	26.8 m	β¯ (100)	295.2228	18.42
				**351.9321**	35.60
	^214^Bi	19.9 m	α (0.021);	**609.320**	45.49
			β¯ (99.979)	1120.294	14.92
				1764.491	15.30
^232^Th	^212^Pb	10.64 h	β¯ (100)	**238.632**	43.6
	^208^Tl	3.053 m	β¯ (100)	510.77	22.60
				**583.187**	85.0
				860.557	12.50
	^228^Ac	6.15 h	β¯ (100)	338.320	11.27
				**911.204**	25.8
				968.971	15.8
^40^K	^40^K	1.248×10^9^ y	EC 10.719);	**1460.822**	10.66
			β+ (0.001);		
			β¯ (89.28)		

The γ-lines in bold were used in activity determination.

### Metals detection

In this study, Inductively Coupled Plasma-Mass Spectrometry (ICP-MS) (Agilent Technologies 7500 Series, USA) was used to determine the metal concentrations. For elemental analysis with ICP-MS, it is necessary to digest the sample completely. About 0.5 g of eachpowdered fish sample was digested in microwave digestion system (Multiwave 3000, PerkinElmer, USA) using a mixture of 5 ml of 65% concentrated HNO_3_ (SpectrosoL grade) and 3 ml of H_2_O_2_ (30%) for 10 minutes using microwave heating. After digestion the samples solution were cooled in a water bath, filtered and brought up to a volume of 50 ml using ultra-pure water (18.2 MΩ-cm) in a volumetric flask.In all cases, 1 blank solution and 5 standards were run with the same reagents used under the same conditions to control possible contamination from digestion procedures.

Calibration of the ICP-MS was performed using multi-element-element calibration standard 2A solution (10 mg/l of each element) prepared by adequate mixing and dilution in 5% HNO of 5 standard solutions (Agilent Technologies, USA, part no. 8500–6940). Concentrations for Na, Mg, Al, K, Ca, Cr, Mn, Fe, Co, Cu, Zn, Cd, As, Se, Rb, Sr, Ba, Hg, Cd, Ni and Pb heavy metals were then determined by using ICP-MS. All the analyses were carried out in several times. The recovered values of all the metals ranged from 88% to 96% of the certified value. The ICP-MS detection limits for Na, Mg, Al, K, Ca, Cr, Mn, Co, Ni, Cu,Zn, As, Rb, Sr and Se was 0.0002 mg kg^−1^, while for Fe, Cd, Ba and Pb was 0.0001 mg kg^−1^.

### Calculations of activity and other radiation indices

#### Radionuclide specific activity

Direct assessment of ^226^Ra (^238^U) and ^228^Ra (^232^Th) via conventional γ-ray spectrometry is inappropriate due to the associated very low decay rates and absence of any intense characteristic γ-lines. However, since the progeny of ^226^Ra (^238^U) and ^228^Ra (^232^Th) remain in secular equilibrium with the parent, the activity of any such progeny represents the activity of the respective parent, a procedure described by among others, thus we assessed the radionuclide activity following the procedure described in ref. [3].

#### Estimation of uncertainties

The combined uncertainty in each sample activity was estimated by considering the following uncertainties: statistical uncertainty of the γ-ray counting (0.5–10%); uncertainties in the detection efficiency (~4%); uncertainties in sample weight (~1.5%), and; uncertainty in γ-ray intensity (~1%). These individual contributions, considered to be independent, were added in quadrature to obtain total uncertainties in the range 4.4–10.9%. The assessed radioactivities, together with their uncertainties, are presented in Table 3.

**Table 3:** Activity concentration (Bq kg^–1^) of ^226^Ra, ^232^Th and ^40^K in marine fish and water in the Strait sof Malacca.

Sample code	Radioactivity concentration of fish (Bq kg^-1^)			Sample code	Radioactivity concentration of fish (Bq kg^-1^)		
	^226^Ra	^232^Th	^40^K		^226^Ra	^232^Th	^40^K
Fi_Bl-1	7.79±0.46	6.06±0.36	387.6±18.8	Wa_Bl-1	1.03±0.12	0.25±0.04	15.2±1.4
Fi_Bl-2	8.16±1.08	6.25±0.65	374.1±20.7	Wa_Bl-2	1.05±0.13	0.27±0.06	17.3±1.5
Fi_Bl-3	7.54±0.79	6.31±0.58	316.0±17.0	Wa_Bl-3	1.08±0.14	0.32±0.07	16.7±1.5
Mean	**7.83**±**0.78**	**6.21**±**0.53**	**359.2**±**18.8**	**-**	**1.05**±**0.13**	**0.28**±**0.06**	**16.4**±**1.5**
Fi_Kl-1	6.32±0.56	3.75±0.34	371.5±19.2	Wa_Kl-1	1.02±0.16	0.34±0.07	16.0±1.3
Fi_Kl-2	6.72±0.72	3.79±0.38	442.4±22.6	Wa_Kl-2	1.41±0.19	0.37±0.08	14.5±1.2
Fi_Kl-3	6.40±0.70	3.58±0.37	382.6±18.9	Wa_Kl-3	1.25±0.18	0.35±0.08	14.8±1.2
Mean	**6.48±0.66**	**3.71±0.36**	**398.9±20.2**	**-**	**1.23±0.17**	**0.35±0.08**	**14.8±1.3**
Fi_Re-1	4.37±0.46	1.62±0.21	268.7±13.8	Wa_Re-1	1.28±0.19	0.26±0.06	20.1±1.7
Fi_Re-2	3.77±0.47	1.92±0.21	299.1±15.1	Wa_Re-2	1.03±0.12	0.30±0.07	20.1±1.6
Fi_Re-3	4.02±0.51	2.25±0.30	296.6±15.5	Wa_Re-3	1.22±0.16	0.25±0.09	19.5±1.7
Mean	**4.05±0.48**	**1.93±0.24**	**288.1±14.8**	**-**	**1.18±0.16**	**0.27±0.07**	**19.9±1.7**
Over all mean	**6.31±0.65**	**4.13±0.39**	**348.7±18.0**	**-**	**1.15±0.15**	**0.30±0.07**	**17.0±1.5**

Sample codes Fi-Bl, Fi-Kl and Fi-Re relate to fish (Ikan Kembung) obtaianed off Bagan Lalang, Port Klang and Pantai Remis respectively, while the respective codes when prefixed by the letters Wa refer to water samples.

#### Daily intake of radioactivities

The daily intake of radioactivity from consumption of fish as the dominant diet, is assumed to acrue from an accumulation of the naturally occuring radionuclides ^226^Ra, ^232^Th and ^40^K in *Rastrelliger kanagurta*. Taking into account the annual marine fish landings of 1,472,240 tonnes (1 short ton = 907.188 kg) in 2012 in peninsular Malaysia [33] and a total adult population of 19.15 million in 2012 [34], the per capita daily intake of natural radionuclides via the consumption of seafood has been calculated using the equation [3,35] and presented in Table 4;

$${\text{D}}_{\mathrm{int}}=\frac{{\text{A}}_{s}\times {\text{A}}_{p}\times {\text{F}}_{c}}{{\text{M}}_{p}\times \text{3}65}$$ (1)

where, *D*_int_ is the daily intake of radioactivities (Bq) by individuals, *A*_s_ is the specific activity of radionuclides of interest (Bq kg^−1^ dry weight), *A*_P_ is the annual production, *F*_c_ is the real fraction consumed, the average value of *F*_c_ being considered as 68% (after a consideration of 32% wastage and export) [36], *M*_P_ is the Malaysian population and 365 indicates the days/year.

**Table 4:** Annual effective dose (μSv y^-1^) and life time cancer risk due to the comsumption of natural radionuclide from the marine fish.

Sample code	Daily intake (Bq d^-1^)			Effective dose (μSv y^-1^)			Total effective dose (μSv y^-1^)	Lifetime cancer risk (LCR)		
	^226^Ra	^232^Th	^40^K	^226^Ra	^232^Th	^40^K		^226^Ra	^232^Th	^40^K
Fi_Bl-1	1.01	0.79	50.33	103.4	66.07	113.9	283.4	2.5×10^−4^	4.9×10^−5^	7.6×10^−4^
Fi_Bl-2	1.06	0.81	48.58	108.3	68.14	109.9	286.4	2.6×10^−4^	5.1×10^−5^	7.3×10^−4^
Fi_Bl-3	0.98	0.82	41.04	100.1	68.79	92.87	261.7	2.4×10^−4^	5.1×10^−5^	6.2×10^−4^
Mean	**1.02**	**0.81**	**46.65**	**103.9**	**67.67**	**105.6**	**277.2**	**2.5**×**10** ^**−4**^	**5.0**×**10** ^**−5**^	**7.0**×**10** ^**−4**^
Fi_Kl-1	0.82	0.49	48.25	83.88	40.88	109.2	234.0	2.0×10^−4^	3.0×10^−5^	7.3×10^−4^
Fi_Kl-2	0.87	0.49	57.45	89.19	41.32	130.0	260.5	2.1×10^−4^	3.1×10^−5^	8.6×10^−4^
Fi_Kl-3	0.83	0.46	49.69	84.94	39.03	112.5	236.4	2.0×10^−4^	2.9×10^−5^	7.5×10^−4^
Mean	**0.84**	**0.48**	**51.80**	**86.00**	**40.41**	**117.2**	**243.6**	**2.1**×**10** ^**−4**^	**3.0**×**10** ^**−5**^	**7.8**×**10** ^**−4**^
Fi_Re-1	0.57	0.21	34.89	58.00	17.66	78.96	154.6	1.4×10^−4^	1.3×10^−5^	5.3×10^−4^
Fi_Re-2	0.49	0.25	38.84	50.04	20.93	87.90	158.9	1.2×10^−4^	1.6×10^−5^	5.8×10^−4^
Fi_Re-3	0.52	0.29	38.52	53.35	24.53	87.16	165.0	1.3×10^−4^	1.8×10^−5^	5.8×10^−4^
Mean	**0.53**	**0.25**	**37.42**	**53.80**	**21.04**	**84.67**	**159.5**	**1.3**×**10** ^**−4**^	**1.6**×**10** ^**−5**^	**5.6**×**10** ^**−4**^
World average	-	-	-	120	120	170	290	-	-	-

The meaning of the sample codes are as in Table 2 above.

#### Committed dose from annual intakes

Estimation of radiation induced health effects associated with the intake of radionuclides are proportional to the total dose delivered by the radionuclides. The committed effective dose to an individual from an intake of a radionuclide via ingestion of one type of food has been calculated by the following formula [37, 38],

$$D_{eff}=A_{s}\times A_{if}\times D_{cf}$$ (2)

where, *D*_eff_ is the annual effective dose to an individual (μSv yr^−1^); *A*_s_ is the specific activities of radionuclides (Bq kg^−1^); *A*_if_ is the annual intake of food (kg yr^−1^), where as mentioned in *section 2*.*5*.*3*, the per capita seafish consumption for Malaysia for the year 2012 was 47.4 kg yr^−1^; and *D*_cf_ is the ingestion dose conversion factor (2.8×10^−7^ Sv Bq^−1^ for ^226^Ra, 2.3×10^−7^ Sv Bq^−1^ for ^232^Th, 6.2×10^−9^ Sv Bq^−1^ for ^40^K) are taken from refs. [1, 39, 40]. The total dose (committed) via ingestion can be calculated using the following formula:

$${\text{D}}_{\text{eff}}^{\text{total}}=[(A_{Ra-226}\times D_{cf})+(A_{Th-232}\times D_{cf})+(A_{K-40}\times D_{cf})]\times (A_{if}\times F_{c})$$ (3)

#### Daily intake of metals (DIM)

The daily intake of metals (Cd, Co, Cr, Mn, Fe, Ni, Cu, Zn, and Pb) depends both on the metal concentration level and the amount of consumption. The *DIM* for adults was estimated using the following equation [41]:

$$\text{DIM}=\frac{{\text{C}}_{metal}\times W}{m}$$ (4)

where *C*_metal_ is the concentration of heavy metals in fish; *W* represents the daily average consumption of fish (130 g) and*m* is the adults body weight (70 kg).

#### Carcinogenic risk

With longevity contributing to greater radiation exposure and by association increasing cancer incidence, an effort was made to assess the lifetime cancer risk due to the ingestion of marine fish by the procedure proposed by the United States Environmental Protection Agency, USEPA [42]. The following equation [37, 43] was used to calculate the mortality cancer risk and is shown in Table 4.

$$LCR=A_{ir}\times A_{ls}\times R_{c}$$ (5)

where *LCR*, *A*_ir_, *A*_ls_ and *R*_c_ are the lifetime cancer risk, annual intake of radionuclide (Bq), average span of life (70 y) and mortality risk coefficient (Bq^–1^), respectively. The values of mortality cancer risk coefficients included 9.56 × 10^−9^ (Bq^–1^) for ^226^Ra, 2.45× 10^−9^ (Bq^–1^) for ^232^Th and 5.89 × 10^−10^ (Bq^–1^) for ^40^K, taken from the USEPA (1999) [42].

#### Statistical analysis

The data were statistically analyzed using SPSS 21 software (IBM Corporation, Armonk, NY, USA). Activity concentrations, daily intake and annual effective dose (using the fish samples data from the three study locations) were compared employing oneway analysis of variance (ANOVA). Post Hoc Tukey HSD test was conducted to verify statistically significant differences among individual means at p < 0.05.

## Results and Discussion

### Radioactivity concentrations

In regard to the natural radionuclides, ^228^Ra (^232^Th) concentration in sea water to be consistently lower than that of ^226^Ra (^238^U). The result supports the solubility of uranium and low-solubility of thorium in water [3, 44], offset to an extent by the greater abundance of thorium in the earth’s crust.In turn, the accumulation of thorium in sea fish can also be expected to be somewhat lower than that of the unranium chain nuclides; the dry weight basis activity concentrations of the investigated radionuclides in fish, and of that in water samples, along with their uncertainties, are summarized in Table 3. Present analysis shows greater concentrations of ^226^Ra than that of ^232^Th in the fish for all three study areas. Statistical analysis (ANOVA) also shows significant variation (p < 0.05) in concentrations of ^226^Ra and ^232^Th in the fish samples among the three studied locations. The greatest mean concentration of ^226^Ra (^238^U) was (7.83 ± 0.78 Bq kg^−1^), being that in Bagan Lalang, Negeri Sembilan, while it was found to be least (4.05 ± 0.48 Bq kg^−1^) in Pantai Remis, Perak. Similarly, for ^228^Ra (^232^Th), the greatest mean concentration (6.21 ± 0.53 Bq kg^−1^) was found in Bagan Lalang and lowest (1.93 ± 0.24 Bq kg^−1^) in Pantai Remis. Of further note that the activity concentrations of ^40^K are significantly greater (p < 0.001) than that of the other radionuclides for all study locations (Table 3), the greatest mean activity concentration of ^40^K being 398.6 ± 20.2 Bq kg^−1^, found in fish samples from Port Klang, Selangor and lowest at 288.1 ± 14.8 Bq kg^−1^ in Pantai Remis, Perak. The appreciably greater values for ^40^K are in line with expectation, a considerable fraction of the weight of each sample being accounted for by the fish bones rich as they are in potassium.

From the results (Table 3), it is clear that concentrations of all detected radionuclides in fish samples was found to be greatest in areas surrounding Bagan Lalang. The possible reasons form a complex mix of factors, linked perhaps to the wide varieties of activity (e.g., housing, tourism, power generation plants, petroleum, chemical industries etc.) around the Bagan Lalang area, industrial and urbanization effluents perhaps increasing the concentrations of radionuclides in the marine environment. It is also important to note that here the Straits are rather more narrow than towards the less industrialized Pantai Remis.

In making comparisons with levels elsewhere, the range of specific activity of ^226^Ra, ^232^Th and ^40^K in sea fish samples from the Black Sea Region of Turkey have been reported [22] as 0.06 ± 0.01 to 0.96 ± 0.36, 0.12 ± 0.04 to 1.03 ± 0.15 and 35.04 ± 0.24 to 127.41 ± 2.29 Bq kg^−1^ respectively. A study [45] reported average activities of ^226^Ra, ^232^Th and ^40^K in fish samples (anchovy) from Korea as < 0.049, 0.0381 and 15.45 Bq kg^−1^ respectively. The average concentrations of ^226^Ra and ^232^Th activity in fish samples from Nigeria have been reported [46] as 0.272 Bq kg^−1^ and 0.115 Bq kg^−1^ respectively. The range of activity concentration of ^238^U, ^232^Th and ^40^K in marine fish samples from the Bay of Bengal, off the coast of Bangladesh, have been reported [47] as 0.11–1.94, 0.24–2.28 and 4.93–77.09, respectively. In all of these cases, the results are very much lower than that of present observations. Conversely, Ariffin et al. 2011 [48] have reported average activity ranges for ^226^Ra and ^228^Ra in the soft tissue of fish collected from Kapar, Klang, Malaysia (near to Port Klang and very near to the 2420 MW Kapar coal-fired power plant) of 11.82 ± 5.23 to 16.53 ± 6.53 Bq kg^−1^ and 43.52 ± 16.34 to 53.57 ± 19.86 Bq kg^−1^ respectively, being considerably greater than the present study.

The estimated daily intake of radionuclides and annual effective doses due to an intake of ^226^Ra (^238^U), ^228^Ra (^232^Th) and ^40^K from the consumption of *Rastrelliger kanagurta*are presented in Table 4. On average, the daily intake of each radionuclide from *Rastrelliger kanagurta* in all study regions were estimated to be 0.80,0.51 and 45.29 Bq for ^226^Ra,^232^Th and ^40^K, respectively.

By ANOVA analysis, the daily intake of ^40^K was found to be significantly greater (p < 0.001) than that of the other radionuclides. The intakes of ^226^Ra (1.7%), ^232^Th (1.1%) are negligible compared with that of the radionuclide ^40^K (96.7%), the latter being a natural isotope of potassium, an essential element for vertebrates and one which is fairly constant as a result of homeostatic regulation [49].

The annual effective dose of ^226^Ra and ^232^Th varies significantly (p < 0.05) among the studied locations. The greatestannual effective dose for ^40^K (117.2μSv y^–1^) was found in Port Klang followed by ^226^Ra (103.9 μSv y^–1^) in Bagan Lalang. Significantly (p < 0.05), the greatest total committed dose was found in Bagan Lalang (278.9 μSv y^–1^), while the least was found in Pantai Remis (160.6 μSv y^–1^). The average worldwide effective dose from the ingestion of uranium and thorium series nuclides is reported to be 120 μSv y^–1^, while for ^40^K it is 170 μSv y^–1^ [30, 36]. The annual effective dose for all detected radionuclides in all study locations are within the [36] mentioned values. The lifetime cancer risk was found to vary from 1.3 × 10^−4^ to 2.5 × 10^−4^ for ^226^Ra, 1.0 × 10^−5^ to 5.0 × 10^−5^ for ^232^Th and 5.6 × 10^−4^ to 7.8 × 10^−4^ for ^40^K, which are low compared with the acceptable cancer risk of 10^−3^ for radiological risk [37,43].

### Metal concentrations

**Fig 4:**
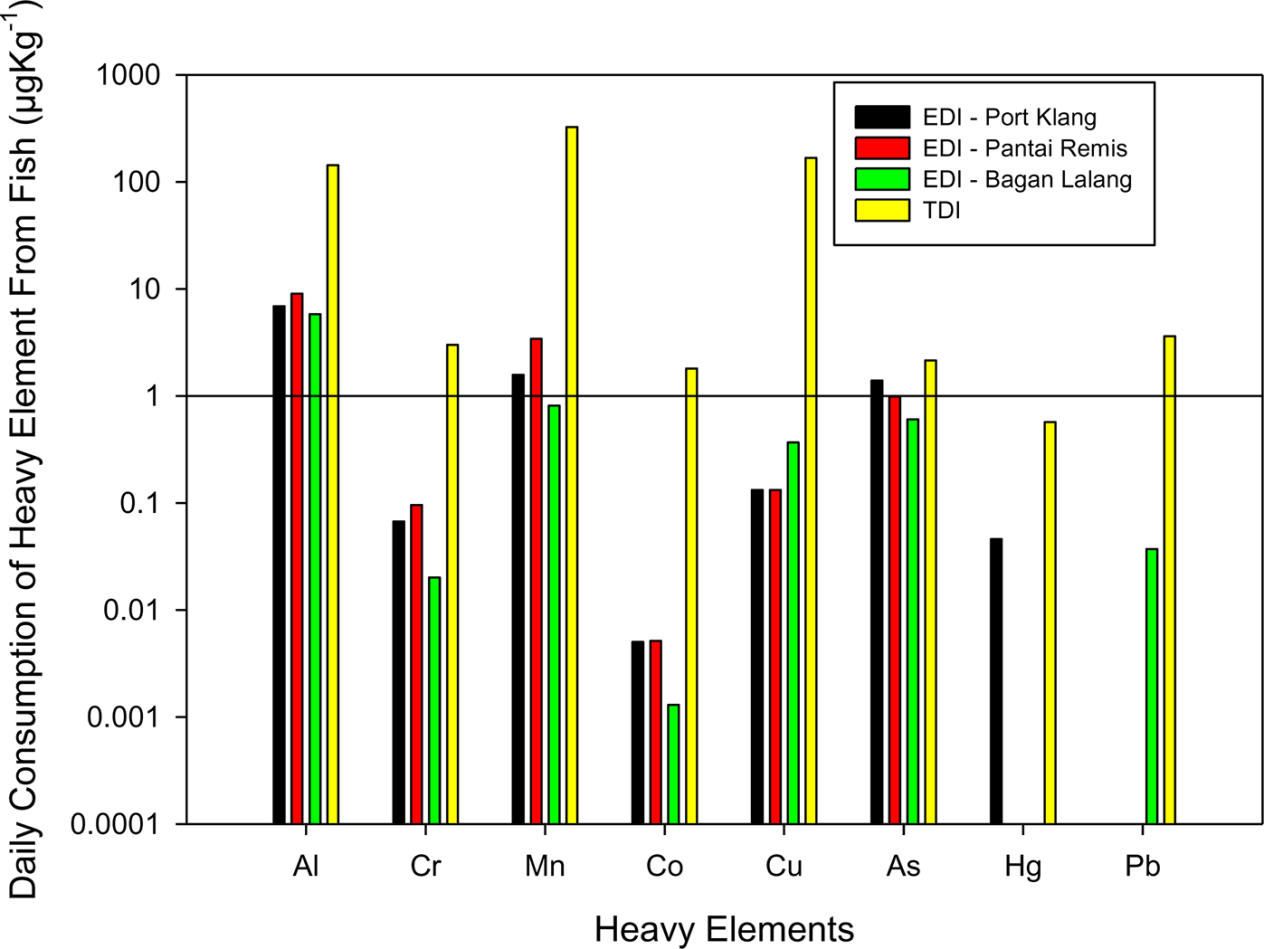
Heavy metal concentrations in fish of Port Klang, Pantai Remis and Bagan Lalang: Comparison of Daily Estimated Intake (EDI) with the Tolarable Daily Intake (TDI) of heavy metals.

The concentrations determined for the metals Al, Cr, Mn, Fe, Co, Cu, Zn, As, As, Hg and Pbin the fish samples are presented in Table 5 and their Estimated Daily Intake (*EDI*) (body weight for 70 kg adults) are reported in Table 6 and in Fig 4.

**Table 5:** Trace element concentrations (mg kg^-1^) in the most frequently consumed sea fish (Ikan Kembung) in Malaysia.

Sample location	Port Klang				PantaiRemis				BaganLalang		
Sample name	Fish-1	Fish-2	Fish-3	Average	Fish-1	Fish-2	Fish-3	Average	Fish-1	Fish-2	Average
Na	233.1	115.1	231	193.1	220.9	219.5	223.5	221.3	260.7	250	255.4
Mg	89.86	49.32	102.1	80.43	87.59	85.54	88.53	87.22	115.4	114.6	115.0
Al	4.488	2.816	3.789	3.698	4.449	4.798	5.301	4.849	3.508	2.722	3.115
K	483.2	290.2	623.4	465.6	464.7	459.7	456.6	460.3	439.4	436.4	437.9
Ca	6899	2684	5592	5058	6980	6487	6675	6714	6614	6241	6427
Cr	0.0432	0.0028	0.0624	0.0361	0.0453	0.0514	0.058	0.0516	0.0108	-	0.0108
Mn	1.985	0.1642	0.3926	0.847	1.942	1.717	1.863	1.841	0.4598	0.4146	0.4372
Fe	4.843	2.102	3.692	3.546	4.796	4.883	4.893	4.857	4.111	3.855	3.983
Co	0.0027	-	-	0.0027	0.0033	0.0012	0.0038	0.0028	0.0007	-	0.0007
Cu	0.0866	0.0314	0.0956	0.0712	0.0683	0.0679	0.0773	0.0712	0.2018	0.1936	0.1977
Zn	17.02	1.888	3.502	7.470	15.17	14.96	15.91	15.35	5.577	5.637	5.607
As	0.6061	0.5523	1.087	0.7485	0.5288	0.5305	0.5393	0.5329	0.3237	0.3262	0.325
Se	0.0471	0.0311	0.0759	0.0514	0.034	0.0332	0.0379	0.035	0.0907	0.0969	0.0938
Rb	0.2428	0.0647	0.1603	0.1559	0.2314	0.2298	0.2325	0.2312	0.098	0.0975	0.0978
Sr	28.2	11.41	23.46	21.02	28.24	26.88	27.93	27.68	20.22	18.95	19.56
Mo	0.5767	0.7037	1.407	0.8958	0.4084	0.3128	0.3034	0.3415	2.719	2.541	2.63
Ba	0.2087	0.256	0.5616	0.3421	0.1845	0.1616	0.5075	0.2845	0.6917	0.6276	0.6597
Bi	8.61	0.4822	3.338	4.143	9.181	1.066	1.821	4.023	2.426	0.7697	1.598
Hg	0.056	0.014	0.004	0.025	-	-	-	-	-	-	-
Pb	-	-	-	-	-	-	-	-	0.02	-	0.02

The average concentration for each metal was used in the calculations of daily intake of metals.

**Table 6:** Estimated daily intake (*EDI*) of heavy metals through consumption of fish.

Sample location	Port Klang		Pantai Remis		Bagan Lalang		TDI (μg/kg/day)
Element	Average Concentration (mg/kg) dry weight	EDI (μg/kg) body weight per day	Average Concentration (mg/kg)	EDI (μg/kg) body weight per day	Average Concentration (mg/kg)	EDI (μg/kg) body weight per day	
Al	3.6977	6.8671	4.8493	9.0059	3.115	5.785	143^a^
Cr	0.0361	0.0671	0.0516	0.0958	0.0108	0.0201	143^b^
Mn	0.8473	1.5735	1.8407	3.4184	0.4372	0.8119	157^c^
Co	0.0027	0.0050	0.0028	0.0051	0.0007	0.0013	20^b^
Cu	0.0712	0.1322	0.0712	0.1322	0.1977	0.3672	142^c^
As	0.7485	1.3900	0.5329	0.9896	0.3249	0.6035	2.14^a^
Hg	0.0247	0.0458	-	-	-	-	0.57^a^
Pb	-	-	-	-	0.02	0.0371	3.60^d^

Sources:

^a^Antoine et al. (2012)

^b^Nutrition^*ATC*^ (2014)

^c^Health Canada, (2007)

^d^Zhuang et al (2009)

A number of national environmental protection agencies report that an intake of 1.0 mg/day of inorganic arsenic (As) is sufficient to give rise to skin lesion after a few years [50]. The greatest mean As concentration observed herein was at Port Klang (0.748 mg/kg dry weight), varying with a factor of about 0.2 for other regions, the least value being found to be (0.325 mg/kg dry weight) at Bagan Lalang. The reported values are appreciably lower than that suggested European Community maximum permissible guideline of 2 mg/kg (dry weight) for marine fish [51] and that reported by Korkmaz Görür e (4.4 mg/kg) of Black Sea fish in Turkey [22].

Mercury (Hg), potentially carcinogenic, can also produce adverse effects during developmental stages as a result of acute or chronic exposure. While there is no known reported safe level [52], the Canadian Food Inspection Agency has set a standard for fish of 0.2 mg/kg dry weight [53]. In present study it has only been detected in fish collected from Port Klang, with values ranging from 0.004 to 0.056mg/kg dry weight, almost certainly reflecting the cummulative activities of many Malaysian industries located in and around the region of the Klang valley. While the level in sampled fish is a factor of 10 below the Tolerable Daily Intake (*TDI*), the estimated daily intake of Hg from consumption of fish does represent a concern worthy of regular monitoring.

Lead (Pb), again a potential carcinogen, can cause adverse health effects [41] including lead poisoning,confusion, poor cognition and kidney damage. In present study, only in one case was Pb was detected, in fish obtained from Bagan Lalang, with a concentration of 0.02 mg/kg, being well below thesuggested level of 2.0 mg/kgin fish [54]. While again this is presently at a level of limited concern, the value serves as an initial datum point from which future changes in levels in the region can be monitored. By way of comparision, [55] reported Pb levels from Turkey ranging from0.09 to 6.95 mg/kg, from fish in the northern Mediterian, while [22] reported a lower range of Pb (< 0.001–0.06 mg/kg) in the fish of the Black Sea of Turkey. The observed large variation between the EDI and TDI suggests Pb levels through consumption of fish is yet below the hamful level.

Chromium (Cr), a proven carcinogenic metal, if taken at a dose of 0.5 mg/kg of body weight per day via oral ingestion [56]. Cr was found with varying concentrations in all samples with the exception of some from Bagan Lalang, with values ranging from 0.003 to 0.052 mg/kg. By way of comparison, the Turkish study of [22] reported Cr at levels of between < 0.1 to 0.73 mg/kg dry weight of fish. Adequate Intake (AI) of 30 μg/kg body weight has been suggested for 70 years adult and above [53]. The results of present study represent a current safe level of exposure.

Elevated manganese (Mn) content showing negative effects on fertility, the central nervous system and embryo and fetal development [57]. In present study, Mn has been detected in all samples at low levels, with a range of 0.4–1.8 mg/kg dry weight, being not too disimilar from that of two Turkish Black Sea studies, at 0.56–1.04 mg/kg [22] and 0.10–0.99 mg/kg of fish [58]. Comparing the upper TDI of this element (324 μg/kg bw) with the calculated EDI (3.4 μg/kg bw), present fish consumption remains at safe level.

Exposure to cobalt (Co) in the bodyorgans has been noted to lead to complicated health problems [59]. Herein, a relatively low concentration of Co has been detected in all of the samples found to be of insignificant risk via TDI and EDI, with a range from a maximum of 0.0028 mg/kg down to 0.0007 mg/kg, from Pantai Ramis and Bagan Lalang respectively, [60] reported a higher range (< 0.05 to 0.30 mg/kg dry weight) of Co in the fish from the coastal region of Turkey around the Black Sea.

Copper (Cu) is essential in all organisms intrace amount, and particularlyserves as a constituent of respiratory enzyme complex in the human body. Due to its role in facilitating iron uptake, deficiency of Cu can lead to impaired growth, anemia-like symptoms, bone abnormalities, and vulnerability to infections. Cu has been found in all of the samples, with concentrations ranging from 0.03 to 0.2 mg/kg dry weight of fish which is below the TDI set by joint experts of FAO/WHO as 3 mg/kg body weight [22] and thelevel of risk is yet insignificant. By way of comparison, reported a range of 0.4 to 1.5 mg/kg from a similar study conducted in Spain [61].

While the health effects of aluminium (Al) has not been widely reported, there is increasing evidence of its toxicity in relation to its gradual accumulation in the brain and subsequent effects on the nervous system [62,63]. In present study, the range of Al content in the sampled fish was 2.7 to 5.3 mg/kg dry weight. The WHO has recently revised its previous Provisional Tolerable Weekly Intake from 7mg/kg body weight to just 1 mg/kg body weight [63]. Comparing this revised WHO value with the estimated values from fish consumption, the current Al level per kg body weight can be considered to remain at a safe level.

### Mineral elements

The marine diet represents a major source of many essential elements, exemplified by present results, with healthy concentrations of iron, zinc, calcium, sodium, magnesium, selenium, etc. The importance of these elements and of deficiency symptoms are well decumented [64], and forms only a minor aspect of this work.

## Conclusions

Owing to their pathway into the daily diet of the local populace, radioactivity and heavy metal concentrations have been assessed in marine life from the Straits of Malacca, west coast of Malaysia. The present investigation shows the radioactivity concentrations of ^226^Ra, ^232^Th and ^40^K in sea water samples in all study sites to be very much lower than that of sea fish samples obtained from the same areas.

Present study shows elevated radioactivity concentrations in the fish of the Straits of Malacca compared to that reported in similar studies in seas elsewhere. The results reflect the contribution of additional technologically enhanced naturally occurring radioactive material (TENORM) pollutants, largely expected to be a result of oil and gas waste streams, related to shipping activities, the route being regarded as the second busiest water channel in the world. In regard to intercomparison of results from the study locations, the Bagan Lalang area showed the greatest level of radioactivity; here, the narrowing of the Straits, together with a relatively high level of industrialisation, urbanization and the effluents that result from these factors are expected to lead to increased concentrations of radionuclides in the marine environment, including fish.

The annual effective doses received by individuals due to the dietary intake of ^226^Ra, ^232^Th and ^40^K via the consumption of fish, range from 154.6 to 286.4 μSv y^–1^ with an average of 226.7 μSv y^–1^, falling below the world average for annual effective dose. Accordingly, the carciogenic risk was found to be well below the acceptable limit of 10^−3^. Present study indicates radionuclide intake from consumption of Straits of Malacca fish poses insignificant threat to public health.

Present study identifies the presence of a wide range of non-essential metals in the selected fish, albeit at relatively low levels compared to studies conducted eleswhere, varying in concentration from region to region and element to element. While again the variation is to be linked with industrial locations and types, the pattern does not agree with that found for NORM nuclides, with levels at Pantai Remis now typically recording the greater levels. Although there may not be internationally agreed safe levels for all of these metals, the overall contamination from the samples would seem to strongly indicate the samples to be well below harmful levels.

## References

[1] UNSCEAR (United Nations Scientific Committee on the effects of Atomic Radiation), Sources and effects of Ionizing radiation. Exposures of the public and workers from various sources of radiation. Report to the General Assembly with Scientific Annexes, Annex-B (2008).

[2] Amin YM, Mahat RH, Nor RM, Khandaker MU, Takleef GH, Bradley DA. The presence of natural radioactivity and ^137^Cs in the South China sea bordering Peninsular Malaysia. Radiat Prot Dosim. 2013;156: 475–480. 10.1093/rpd/nct097 23584496

[3] Khandaker MU, Norfadira BW, Amin YM, Bradley DA. Committed effective dose from naturally occurring radionuclides in shellfish. Radiat Phys Chem. 2013;88: 1–6.

[4] Abbasisiar FT. Hosseini A, Heravi FG. Determination of uranium isotopes (^234^U, ^238^U) and natural uranium (U-nat) in water samples by alpha spectrometry. Iran J Radiat Res. 2004; 2: 1–6.

[5] Khan MF, Benjamin J, Godwin SW. Radiotoxicity via intake of marine organisms: exposure and risk assessment in South Indians. Toxicol Environ Chem. 2011;93: 549–564.

[6] IAEA TECDOC-1105 “Inventory of radioactive waste disposals at sea”. Available at http://www-pub.iaea.org/books/iaeabooks/5786/Inventory-of-Radioactive-Waste-Disposals-at-Sea

[7] Bogatov S, Kisselev V, Sorokovikova O, Vysotsky V. Radiation consequences of hypothetical accidents associated with transportation of spent nuclear fuel of nuclear submarines aboard floating technical base. Radioprotection 2009;44(5), 159–164.

[8] Carvalho FP, Oliveira JM, Malta MM. Radionuclides in deep-sea fish and other organisms from the North Atlantic Ocean. ICES J Mar Sci. 2011;68: 333–340.

[9] Fraizier A, Guary JC. Recherche D’indicateurs Biologiques Appropries au Controle de Lacantamination du Littoral par le Plutonium (in English: Search of Biological Indicators Appropriate to Control Contamination in Coastal by Plutonium). IAEA Transuranic Nuclides in the Environment, IAEA-SM-199/18 (1976); 679–89.

[10] Guary JC, Fraizier A. Influence of trophic level and calcifation on the uptake of plutonium observed, in situ, in marine organisms. Health Phy. 1977;32: 21–28. 10.1097/00004032-197701000-00003 838585

[11] Makon TB, Nemba RM. Tchokossa P. Investigation of gamma-emitting natural radioactive contents in three types of Vernonia consumed in Cameroon. World J Nucl Sci Technol. 2011;1: 37–45.

[12] UNSCEAR (United Nations Scientific Committee on the Effects of Atomic Radiation), Ionizing radiation: sources and biological effects. United Nations New York, (1982a).

[13] Real A, Sundell-Bergman S, Knowles JF, Woodhead DS, Zinger I. Effects of ionizing radiation exposure on plants, fish and mammals: relevant data for environmental radiation protection. J Radiol Prot. 2004;24: A123–A137. 15700702 10.1088/0952-4746/24/4a/008

[14] Alina M, Azrina A, Mohd Yunus AS, Mohd Zakiuddin S, Mohd Izuan Effendi H, Muhammad Rizal R. Heavy metals (mercury, arsenic, cadmium, plumbum) in selected marine fish and shellfish along the Straits of Malacca. Int Food Res J. 2012;19: 135–140.

[15] Ahalya N, Ramachandra TV, Kanamadi RD. Biosorption of Heavy Metals. Res J Chem Environ. 2003;7: 71–79.

[16] Khan MF, Wesley SG. Assessment of health safety from ingestion of natural radionuclides in sea foods from a tropical coast India. Mar Pollut Bull. 2011;62: 399–04. 10.1016/j.marpolbul.2010.12.016 21251682

[17] Pentreath RJ. The biological availability to marine organisms of transur- anium and the other long-lived nuclides In. Proceedings of International Symposium on the Impacts of Radionuclide Releases into the Marine Environ- ment. IAEA-SM-248/102, Vienna, October 1980; 241–72.

[18] Blaylock BG. Radionuclide data bases available for bioaccumulation factors for freshwater biota. Nucl Saf. 1982;23: 427–38.

[19] IAEA (International Atomic Energy Agency))Sources of radioactivity in the marine environment and their relative contributions to overall dose assessment from marine radioactivity (MARDOS), IAEA-TECDOC-838, Vienna (1995).

[20] Didem Aydin D, Tokalıoğlu S. Trace metals in tissues of the six most common fish species in the Black Sea, Turkey. Food Additives & Contaminants: Part B. 2015;8: 25–31. 10.1080/19393210.2014.949873 25082436

[21] Freeman DB. The Straits of Malacca: Gateway or Gauntlet? McGill-Queen’s University Press. ISBN 0-7735-2515-7. A book review citing this information can be found at University of Toronto Quarterly, Winter 2004/5 200374; 528–30.

[22] Korkmaz GF, Keser R, Akcay N, Dizman S. Radioactivity and heavy metal concentrations of some commercial fish species consumed in the Black Sea Region of Turkey. Chemosphere. 2012;87: 356–61. 10.1016/j.chemosphere.2011.12.022 22225706

[23] Kalay M, Canli M. Elimination of essential (Cu, Zn) and nonessential (Cd, Pb) metals from tissue of a freshwater fish Tilapia zillii following an uptake protocol, Tukr J Zool 24(2000)429–36.

[24] Fishbein L, Furst A, Mehlman MA. Genotoxic and carcinogenic metals: environmental and occupational occurrence and exposure Princenton Scientific Publishing, USA 1987; pp. 339.

[25] Emilio R, Luis AH, Lionel AP, Patricia OW. Are metals dietary carcinogens? Mutation Res. 1999;44(3): 157–81. 10.1016/s1383-5742(99)00018-6 10415439

[26] Khandaker MU, Olatunji MA, Shuib KSK, Hakimi NA, Nasir NLM, Asaduzzaman Kh, et al. Natural radioactivity and effective dose due to the bottom sea and estuaries marine animals in the coastal waters around Peninsular Malaysia. Radiat Prot Dosim. 2015; pp. 1–5. 10.1093/rpd/ncv243 25956784

[27] Connan O, Germain P, Solier L, Gouret G. Variations of ^210^Po and ^210^Pb in various marine organisms from western English Channel: contribution of ^210^Po to the radiation dose. J Environ Radioact. 2007; 97:168–88. 17566617 10.1016/j.jenvrad.2007.04.004

[28] Hvistendahl M. Coal Ash is More Radioactive than Nuclear Waste. Scientific American 2007.

[29] US Geological SurveyRadioactive Elements in Coal and Fly Ash: Abundance, Forms, and Environmental Significance, Fact Sheet FS-163-1997, 1997.

[30] Asaduzzaman Kh, Khandaker MU, Amin YM, Bradley DA, Mahat RH, Nor RM. Soil-to-root vegetable transfer factors for ^226^Ra, ^232^Th, ^40^K, and ^88^Y in Malaysia. J Environ Radioact. 2014 135: 120–27. 10.1016/j.jenvrad.2014.04.009 24814722

[31] Amin YM, Khandaker MU, Shyen AKS, Mahat RH, Nor RM, Bradley DA. Radionuclide emissions from a coal-fired power plant. Appl Radiat Isot. 2013;80: 109–16. 10.1016/j.apradiso.2013.06.014 23891979

[32] Khandaker MU, Jojo PJ, Kassim HA, Amin YM. Radiometric analysis of construction materials using HPGe gamma-ray spectrometry. Radiat Prot Dosim. (2012a);152: 33–37. 10.1093/rpd/ncs145 22887119

[33] Department of fisheries, malaysia, available at <http://www.dof.gov.my>.

[34] Malaysia people 2014, Source: 2014 CIA world factbook and other sources, available at <http://www.theodora.com/wfbcurrent/malaysia/malaysia_people.html>.

[35] Alam L, Mohamed CAR. Natural radionuclide of Po-210 in the edible seafood affected by coal-fired power plant industry in Kapar coastal area of Malaysia. Environ Health. 2011;10: 43–52. 10.1186/1476-069X-10-43 21595985 PMC3125231

[36] UNSCEAR (United Nations Scientific Committee on the effects of Atomic Radiation), Exposures from natural radiation sources;Annex-B (2000); pp140.

[37] Asaduzzaman Kh, Khandaker MU, Amin YM, Mahat R. Uptake and distribution of natural radioactivity in rice from soil in northand west part of peninsular Malaysia for the estimation of ingestion dose to man. Ann Nucl Energy. 2015;76: 85–93.

[38] Ghose S, Alam MN, Islam MN. Radiation dose estimation from the analysis of radionuclides in marine fish of the bay of bengal, Radiat Prot Dosim. 2000;87: 287–91.

[39] ICRP (International Commission on Radiological Protection), Age-Dependent Doses to Members of the Public From Intake of Radionuclides: Part 2. Ingestion Dose Coefficients. ICRP Publication 67. Pergamon Press. Oxford, Annals of the ICRP 23(3/4) (1994). 7978694

[40] IAEA International Basic Safety Standards for Protection against Ionizing Radiation and for the Safety of Radiation Sources Safety Series No. 115. International Atomic Energy Agency, Vienna (1996).

[41] Zhuang P, McBride MB, Xia H, Li N, Li Z. Health risk from heavy metals via consumption of food crops in the vicinity of Dabaoshan mine, South China. Sci Total Environ. 2009; 407: 1551–61. 10.1016/j.scitotenv.2008.10.061 19068266

[42] US Energy Protection Agency (USEPA). Cancer risk coefficients for environmental exposureto radionuclides. Federal Guidance Report No.13;EPA 402-R-99-001 (1999).

[43] Patra AC, Mohapatra S, Sahoo SK, Lenka P, Dubey JS, Tripathi RM, et al. Age-dependent dose and health risk due to intake of uranium in drinking water from Jaduguda, India. Radiat Prot Dosim. 2013 1–7. Radiation Protection Dosimetry Advance Access published March 22, 2013. 10.1093/rpd/ncs328 23525912

[44] Hyde EK. The Radiochemistry of Thorium Subcommittee on Radiochem- Cistry, National Academy of Sciences-National Research Council (1960); available at <http://www.radiochemistry.org/periodictable/pdf_books/pdf/rc000034.pdfS>.

[45] Choi MS, Lin XJ, Lee SA, Kim W, Kang HD, Doh SH, et al Daily intakes of naturally occurring radioisotopes in typical Korean foods. J Environ Radioact. 2008;99: 319–23. 10.1016/j.jenvrad.2008.04.003 18490085

[46] Aragunjo AM, Hollriegle V, Giussani A, Leopold K, Gelrstmann U, Veronese I, et al Uranium and thorium in soils, mineral sands, water and foodsamples in a tin mining area in Nigeria with elevated activity. J Environ Radioact. 2009;100: 232–40. 10.1016/j.jenvrad.2008.12.004 19147259

[47] Alam MN, Chowdhury MI, Kamal M, Ghose S. Radioactivity in Marine Fish of the Bay of Bengal. Appl Radiat Isot. 1995;46: 363–64. 7581291 10.1016/0969-8043(95)00013-4

[48] Ariffin NAN, Mahmood ZUW, Mohamed CAR. Application of in-House Method for Determination of Radium Isotopes in Environmental Samples Using the Liquid Scintillation Counting. J Anal Sci Meth Instrum.2011;1: 1–8.

[49] Awudu AR, Faanu A, Darko EO, Emi-Reynolds G, Adukpo OK, Kpeglo DO, et al Preliminary studies on ^226^Ra, ^228^Ra, ^228Th^ and ^40^K concentrations in foodstuffs consumed by inhabitants of Accra metropolitan area, Ghana. J Radioanal Nucl Chem. 2012;291: 635–41.

[50] Roychowdhury T, Tokunaga H, Ando M. Survey of arsenic and other heavy metals in food composites and drinking water and estimation of dietary intake by the villagers from an arsenic-affected area of West Bengal, India. Sci Total Environ. 2003;308: 15–35. 12738198 10.1016/S0048-9697(02)00612-5

[51] EEC. Setting maximum levels for certain contaminants in foodstuffs. Commission Regulation (EC) No 466/2001of 8 March 2001. Official Journal of the European Communities; 2001:L77.

[52] Bose-O’ Reilly S, McCarty KM, Steckling N, Lettmeier B. Mercury Exposure and Children’s Health, Current Problems in Pediatric and Adolescent. Health Care. 2010;40: 186–15. 10.1016/j.cppeds.2010.07.002 PMC3096006 20816346

[53] Health Canada. Human Health Risk Assessment of Mercury in Fish and Health Benefits of Fish Consumption. Bureau of Chemical Safety Food Directorate Health Products and Food Branch; 2007. http://www.hc-sc.gc.ca/fn-an/alt_formats/hpfb-dgpsa/pdf/nutrition/merc_fish_poisson-eng.pdf

[54] WHO. Guidelines for drinking water quality (2nd ed.). Chemical aspects. Geneva: WHO,1996 <http://www.who.int/water_sanitation_health/dwq/gdwq2v1/en/> (accessed 10.05.10.).

[55] Türkmen A, Türkmen M, Tepe Y, Akyurt I. Heavy metals in three commercially valuable fish species from Iskenderun Bay, Northern East Mediterranean Sea, Turkey. Food Chem. 2005;91: 167–72.

[56] Stern A. Quantitative assessment of the carcinogenicity of hexavalent chromium by the oral route and its relevance to human exposure. Environ Res. 2010;110: 798–07. 10.1016/j.envres.2010.08.002 20843510

[57] Gerber GB, Léonard A, Hantson P. Carcinogenicity, mutagenicity and teratogenicity of manganese compounds. Critical Reviews in Oncology/Hematology 2002;42: 25–34. 11923066 10.1016/s1040-8428(01)00178-0

[58] Tuzen M. Toxic and essential trace elemental contents in fish species from the Black Sea, Turkey. Food Chem Toxicol. 2009;47: 1785–90. 10.1016/j.fct.2009.04.029 19406195

[59] De Boeck M, Kirsch-Volders M, Lison D. Cobalt and antimony: genotoxicity and carcinogenicity. Mutation Research/Fundamental and Molecular Mechanisms of Mutagenesis 2003;533: 135–52. 14643417 10.1016/j.mrfmmm.2003.07.012

[60] Topcuoğlu S, Kırbaşoğlu Ç, Güngör N. Heavy metals in organisms and sediments from Turkish Coast of the Black Sea 1997–1998. Environ Int. 2002;27: 521–26. 11868661 10.1016/s0160-4120(01)00099-x

[61] Usero J, Izquierdo C, Morillo J, Gracia I. Heavy metals in fish (Solea vulgaris, Anguilla anguilla and Liza aurata) from salt marshes on the southern Atlantic coast of Spain. Environ Int. 2003;29: 949–56. 10.1016/S0160-4120(03)00061-8 14592572

[62] Mir-Marqués A, Cervera ML, de la Guardia M. A preliminary approach to mineral intake in the Spanish diet established from analysis of the composition of university canteen menus. J Food Compos Anal. 2012;27: 160–68.

[63] Antoine JMR, Hoo Fung LA, Grant CN, Dennis HT, Lalor GC. Dietary intake of minerals and trace elements in rice on the Jamaican market. J Food Compos Anal. 2012;26: 111–21.

[64] FAO. Human Vitamin and Mineral Requirements. Report of a Joint FAO/WHO Expert Consultation, Bangkok, Thailand. Food and Nutrition Division, FAO Rome; 2001.

